# Rapid progression of subcutaneous glioblastoma: A case report and literature review

**DOI:** 10.3389/fonc.2023.935944

**Published:** 2023-01-25

**Authors:** Fang Wang, Jiawei Dong, Jiheng Zhang, Hongtao Zhao, Nan Wang, Jiaqi Jin, Xiuwei Yan, Xin Gao, Han Liu, Shaoshan Hu

**Affiliations:** ^1^ Cancer Center, Department of Neurosurgery, Zhejiang Provincial People's Hospital, Affiliated People's Hospital, Hangzhou Medical College, Hangzhou, Zhejiang, China; ^2^ Department of Neurosurgery, The Second Affiliated Hospital of Harbin Medical University, Harbin, Heilongjiang, China; ^3^ Department of Neurology, The First Affiliated Hospital of Zhengzhou University, Zhengzhou, Henan, China

**Keywords:** glioblastoma, extracranial metastasis, invade, scalp, case report

## Abstract

Extra-neural spread of glioblastoma (GBM) is extremely rare. We report a case of postoperative intracranial GBM spreading to the subcutaneous tissue *via* the channel of craniotomy defect in a 73-year-old woman. Radiological images and histopathology indicate that the tumor microenvironment of the subcutaneous tumor is clearly different from the intracranial tumor. We also model the invasion of GBM cells through the dura-skull defect in mouse. The retrospective analysis of GBM with scalp metastases suggests that craniectomy is a direct cause of subcutaneous metastasis in patients with GBM. Imaging examinations of other sites for systemic screening is also recommended to look for metastases outside the brain when GBM invades the scalp or metastasizes to it.

## Introduction

Glioblastoma (GBM) is the most common and lethal malignant intracranial tumor, with a median survival of 14.6 months (1). Nonetheless, extra-neural metastases of GBM are considered rare, with a reported incidence of about 0.4%–0.5% (2). Literatures reported that the most frequent sites of extra-neural metastases include lungs, pleura, lymph nodes, liver, and bone, but scalp involvement (directly spread/metastatic seeding) is extremely rare (3).

In this report, we present one patient with intracranial GBM spreading to subcutaneous tissue *via* the channel of craniotomy defect. Subsequently, the histopathological characteristics of extracranial tumors are compared with intracranial tumors based on patient tissue samples and animal experiments. Furthermore, GBM with scalp involvement are also reviewed in this article.

## Methods

### Clinical information and samples of patient

All information was obtained from patient and clinical records. Multi-region sampling of the tumor was performed during the operation under written consent of the patient’s custodians. All clinical information and samples of the patient were shown and handled in accordance with local ethical and legal standards.

### Animal model

Female C57BL/6 mice (13 to 15 months old) were purchased from Vital River Experimental Animal Center (Beijing, China). Mice were anesthetized and fixed in a stereotactic frame (RWD, Shenzhen, China). The scalp was incised and a 1-mm-diameter hole was drilled through the skull. Stereotactic technique was used to implant GL261 cells (5 × 10^4^ cells/mouse) into the right brain (antero-posterior = 1.0 mm; medio-lateral = 2.0 mm; depth = 2.5 mm). Mice were monitored daily and euthanized at moribund state, or at day 21 post-injection. A total of 14 mice were used for making the orthotopic glioma model. At the animal experimental endpoint, extracranial tumor formation was observed in nine mice. The whole brains and the extracranial tumor were removed and fixed with 4% paraformaldehyde and embedded in paraffin for subsequent analysis. The experimental protocol was approved by the Animal Ethical and Welfare Committee of Zhejiang Provincial People’s Hospital.

### Quantitative immunohistochemistry

Immune complexes were detected with the SP Kit (Solarbio, Beijing, China) and DAB Substrate Kit (Solarbio, Beijing, China). Signals were detected using an Olympus BX41 microscope. Images were magnified to ×40 and analyzed using the IHC profiler as previously described (4, 5). Quantification of IHC staining was performed in a blinded fashion for all experiments.

## Case report

A 73-year-old woman presented with 1 month history of headache and dizziness. The patient presented here had no history of psychiatric or head trauma, and reported no relevant family history of cancer. Computed tomography (CT) scan revealed a space-occupying lesion in the right temporal–occipital lobe, surrounded by significant edema (**Supplementary Figure 1A**). A high-grade glioma was suspected; then, she underwent lesion resection at a local hospital. Postoperative magnetic resonance imaging (MRI) showed complete resection of the lesion (day 3 after surgery) (**Supplementary Figure 1B**). The histopathological analysis confirmed the diagnosis of GBM. Tumor tissue showed pseudo-palisading necrosis and microvascular proliferation (**Supplementary Figure 1C**). Immunohistochemistry (IHC) revealed positive staining for glial fibrillary acidic protein (GFAP), CD-34, S-100, and P53 (40%), but negative for O-6-methylguanine-DNA methyltransferase (MGMT) and isocitrate dehydrogenase (IDH). The patient refused concurrent chemoradiation and other further treatments.

**Figure 1:**
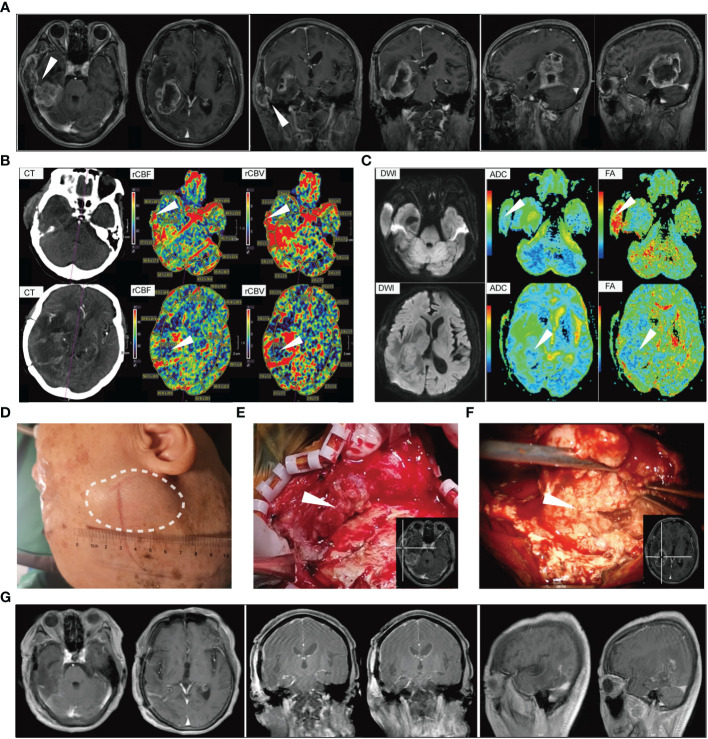
The serial perioperative imaging and tumor characteristics at second surgery. **(A)** The preoperative magnetic resonance imaging. White arrows indicate that the extracranial tumor is directly connected to the intracranial tumor *via* the channel of craniotomy defect. **(B)** The relative cerebral blood flow/volume maps based on CT perfusion images. White arrows indicate that the blood supply was significantly higher in the core region of extracranial tumor compared to the core region of intracranial tumor. **(C)** Corresponding apparent diffusion coefficient (ADC) and fractional anisotropy (FA) maps of the patient. **(D)** The scalp lesion before resection. **(E)** Representative image of the subcutaneous tumor. The white arrow indicates the core region of extracranial tumor. **(F)** Intraoperative picture under a surgical microscope. The white arrow indicates the core region of the intracranial tumor. **(G)** The postoperative magnetic resonance imaging (1 week after surgery).

Two months after the initial surgery, the patient noticed a mass under the right temporal scalp, and the mass had rapidly grown in size within 1 month. Then, she presented to our hospital, 3 months after original diagnosis. MRI showed tumor local recurrence and invasion to subcutaneous tissue *via* the channel of craniotomy defect (**Figure 1A**). The relative cerebral blood volume (rCBV) and relative cerebral blood flow (rCBF) maps acquired from CT perfusion indicated that the blood supply was significantly higher in the core region of the extracranial tumor compared to the core region of the intracranial tumor (**Figure 1B**). **Figure 1C** shows the corresponding apparent diffusion coefficient (ADC) and fractional anisotropy (FA) maps of the tumor area. The ADC/FA maps showed that the ADC value at the intracranial tumor central portion was higher than that at the extracranial region, and the FA value at the intracranial tumor central portion was lower than that at the extracranial region. No abnormal findings were observed during the chest CT scan and abdominal ultrasound examination. Then, the patient underwent reoperation. The extracranial mass is localized under the temporal scalp, and closely attached to the temporalis (**Figures 1D, E**). Under the light microscope, the intracranial tumor is roughly fish flesh-like, with a rich blood supply (**Figure 1F**). Multi-region (*n* = 3) sampling was performed at the core region of the intracranial and extracranial tumor, respectively. Postoperative MRI showed a complete resection of the tumor (1 week after surgery) (**Figure 1G**). **Supplementary Figure 2** showed the timeline of therapy and disease status of the patient.

## Pathological findings and animal experiments

**Figure 2:**
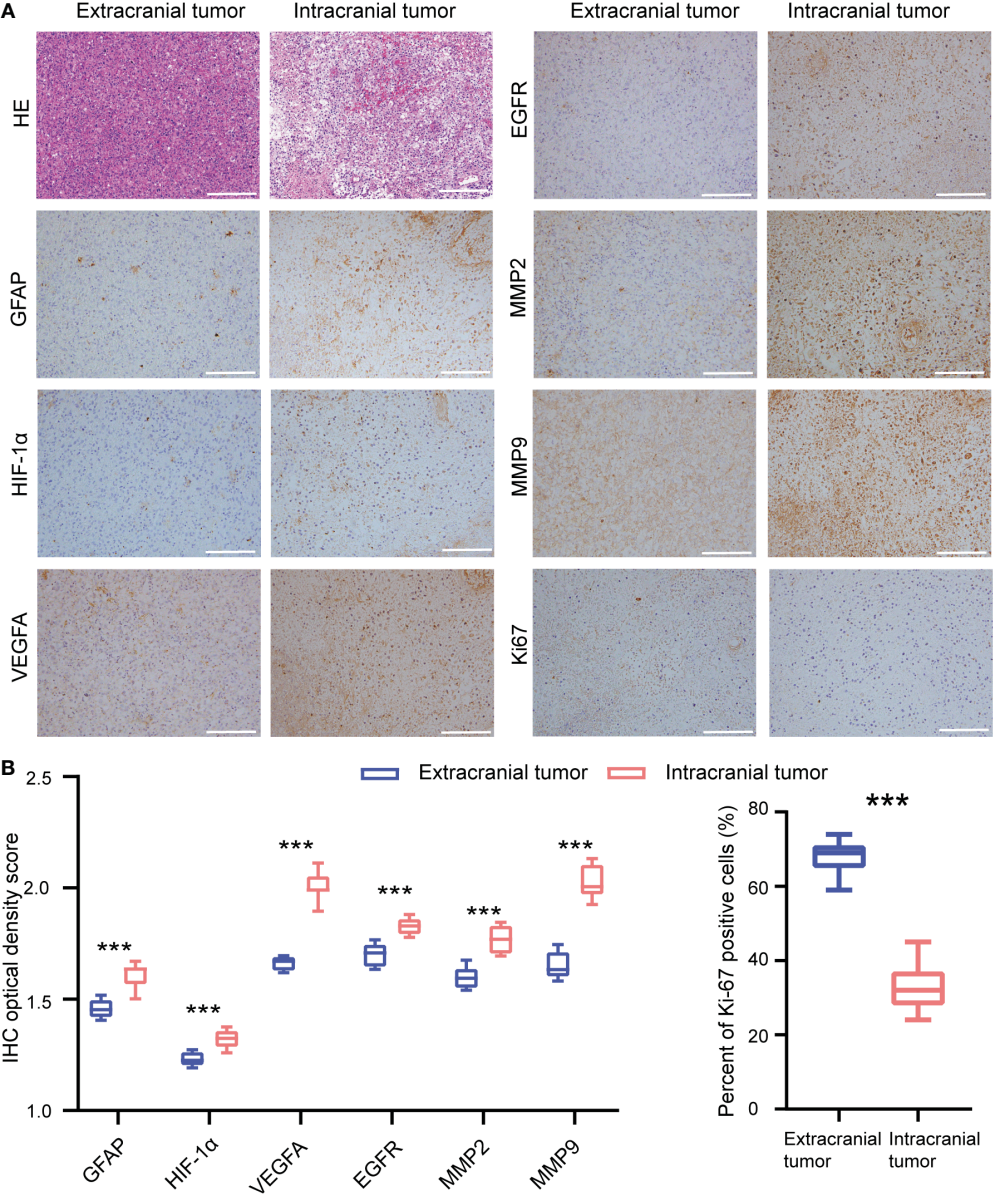
Immunohistochemistry analysis of the extracranial tumor and intracranial tumor. **(A)** Representative immunohistochemical images of extracranial tumor and intracranial tumor in patient. **(B)** Quantification of the immunohistochemical stains. Scale bar = 200 μm. ***p < 0.001.

The histological analysis of both intracranial and extracranial samples confirmed the diagnosis of relapse of GBM. Histopathologic pictures after hematoxylin and eosin (HE) staining of different lesion areas are shown in **Figure 2A**. The signs of pseudo-palisading necrosis could not be seen in the extracranial tumor but are abundant in the intracranial tumor core by HE staining. GFAP-positive cells were rare in the subcutaneous tumor, suggesting that the tumor cellular composition is changed when GBM invades the scalp. Therefore, we further explored spatial heterogeneity in the tumor microenvironment (TME) between them. In addition, IHC results indicated that the expression of hypoxia-inducible factor-1α (HIF-1α), VEGFA, and EGFR in the extracranial tumor are also significantly lower (*p* < 0.001) than in the intracranial tumor (**Figure 2B**). In this study, we found that both MMP2 and MMP9 expressions are significantly higher (*p* < 0.001) in the intracranial tumor (**Figure 2B**). IHC-assessed Ki67 positivity was significantly higher in the extracranial tumor than in the intracranial tumor (67.93 ± 3.99% *vs*. 32.87 ± 5.68%). Collectively, these results indicate that the TME is significantly altered in the extracranial tumor, and tumor cells may have more robust growth under the scalp, most likely due to the rich blood supply existing in that area.

**Figure 3:**
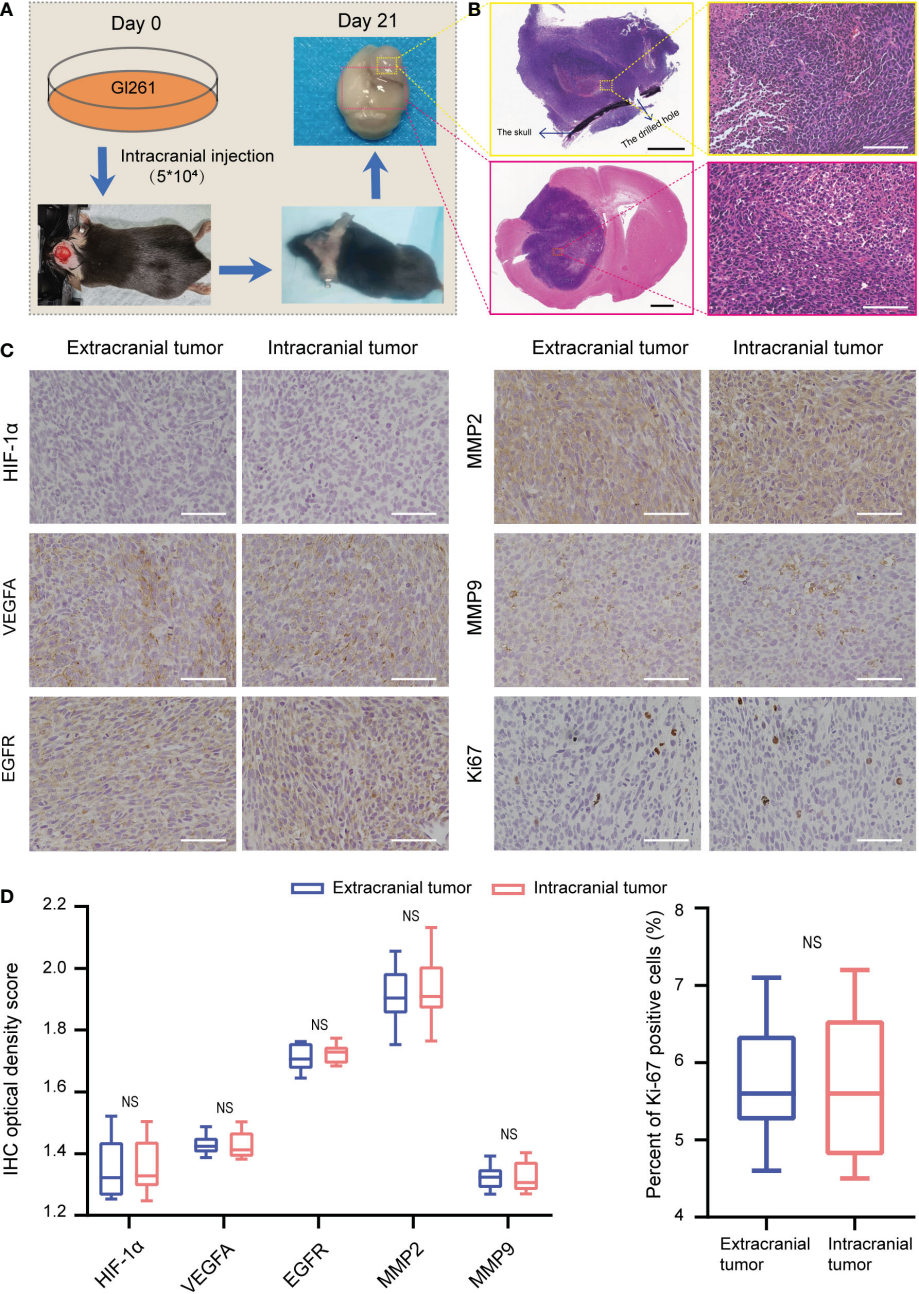
Construction of the mouse model and immunohistochemistry analysis. **(A)** The flowchart shows the process of creating the glioma mouse model. **(B)** Hematoxylin and eosin stains of extracranial tumor and intracranial tumor in mouse. Black scaler bar = 1 mm, white scale bar = 100 μm. **(C, D)** Immunohistochemistry analysis of the extracranial tumor and intracranial tumor in mouse. Scale bar = 100 μm. NS, not significant.

In order to further assess the difference in tumor microenvironment between intracranial and extracranial tumors, further studies utilizing animal models with glioma were performed (**Figure 3A**). HE staining showed that the histological characteristics were indistinguishable between the intracranial tumor and the extracranial tumor (**Figure 3B**). In addition, we also explored the expression of the indicated proteins in different regions. The immunohistochemical results indicated that the extracranial tumor that invades the scalp has a similar TME to the intracranial tumor in the mouse model (**Figures 3C, D**). Nonetheless, they suggest that glioma cells could also spread to subcutaneous space *via* the channel of craniotomy defect in the mouse model.

## Discussion

Although GBM is the most aggressive nervous system cancer, it extremely rarely invades the subcutaneous tissue. Only seven cases of intracranial GBM that spread to the scalp through surgical sites were reported in literatures (6–11). Moreover, there is another scalp metastasis that is not directly connected with the intracranial tumor. As such, scalp metastasis is usually located close to the surgical incision site, and this suggested that direct tumor seeding is a possible mechanism (12). To our knowledge, less than 20 cases of scalp metastases from GBM cells seeding were described (3, 13–25). We summarize GBM patients with scalp involvement to further explore the characteristics of scalp metastasis (**Table 1**).

**Table 1:** Review from the literatures of 24 reported GBM with scalp metastases.

No.	Author and year	Age (years)/sex	Primary location	MGMT methylation	IDH-1/2 mutation	Adjuvant therapy	Interval time 1 (months)	Scalp metastases localization	Connected with intracranial tumor?	Other sites of metastasis	Interval time 2 (months)
1	Xing-Zhao Luan, 2021 (13)	47/M	R temporoparietal occipital	–	–	Chemo/radiation	7	R parietal	No	Bone, liver, and lung	5
2	Clara El Nakib, 2022 (3)	53/M	R internal capsule/thalamus	NS	–	Chemoradiation	11	The front of the R ear	No	NS	4
3	Jing Liu, 2020 (14)	46/M	L temporal	–	–	Chemo/radiation	6	L frontal and temporal	No	Lungs, pleural area, lymph nodes, et al.	14
4	T. Capion, 2019 (6)	55/M	R parieto-occipital	–	–	Chemo/radiation	6	R temporal and parietal	Yes	Lung and lymph nodes	2
5	Dorte Schou Nørøxe, 2019 (15)	62/M	L parietal	–	+	Chemo/radiation	5	L occipital	No	NS	10
6	Natalia Frade Porto, 2019 (16)	72/M	R temporal	NS	–	Chemo/radiation	5	R temporal	No	Temporal muscle, lymph nodes	NS
7	Jordi Pérez-Bovet, 2018 (7)	63/F	R frontoparietal	+	NS	Chemo/radiation	6	R temporal, L parietal	Yes	NS	4
8	Bella Nguyen, 2018 (17)	62/M	L frontal	NS	–	Chemo/radiation	9	Parietal	No	NS	5
9	Tiara M. Forsyth, 2015 (8)	59/F	L frontotemporal	–	–	Chemo/radiation	6	Frontal	Yes	NS	3.5
10	Anghileri Elena, 2016 (9)	30/M	L parietal	–	–	Chemo/radiation	72	Parietal	Yes	Neck, lung	7
11	Anghileri Elena, 2016 (9)	43/M	L frontal	–	NS	Chemo/radiation	23	Parietal	Yes	NS	2
12	Daniel T. Ginata, 2013 (12)	62/M	L frontal	+	NS	Chemo/radiation	10	L frontal	NS	NS	4
13	Ignacio Jusué Torres, 2011 (10)	63/F	R frontal	NS	NS	Chemo/radiation	6	L frontal	Yes	NS	NS
14	Rebecca Senetta, 2009 (18)	48/F	R frontoparietal	NS	NS	Chemo/radiation	14	Parietal	No	NS	3
15	Rebecca Senetta, 2009 (18)	53/F	L frontal	NS	NS	Chemo/radiation	9	L parietal	No	NS	6
16	Mark Mentrikoski, 2008 (19)	58/F	L frontal	NS	NS	Chemo/radiation	16	L frontal	No	NS	NS
17	Ali G. Saad, 2007 (11)	13/M	L frontal	NS	NS	Chemo/radiation	6	L temporalis	Yes	Liver, lung	4
18	Stacey Schultz, 2005 (20)	74/F	L temporal	NS	NS	Radiation	12	L parietal	No	NS	1
19	R. S. Allan, 2004 (21)	60/M	L frontal	NS	NS	Radiation	12	L frontal	No	NS	2
20	N. Jain, 2005 (22)	49/M	R temporoparietal	NS	NS	Radiation	10	R temporoparietal	No	NS	2
21	Patricio Figueroa, 2002 (23)	34/M	L thalamus	NS	NS	Radiation	7	Frontal	No	NS	4
22	Stephen C. Houston, 2000 (24)	19/M	L parietal	NS	NS	Radiation	10	Occipital	No	Supraclavicular node mediastinum	7
23	Stephen C. Houston, 2000 (24)	32/M	L temporal	NS	NS	Chemo/radiation	6	L frontal	No	Lung, liver	7
24	Paulo A. Carvalho, 1991 (25)	26/F	R temporal	NS	NS	Radiation	NS	R temporal	No	NS	NS

MGMT, the O-6-methylguanine-DNA methyltransferase; IDH, isocitrate dehydrogenase; Interval time 1, time from diagnosis of GBM to the discovery of scalp metastasis; Interval time 2, survival after diagnosis of scalp metastasis in months; R, right; L, left; M, male; F, female; NS, not stated or not known.

In this cohort of 24 GBM patients with scalp involvement, the median time from diagnosis of GBM to the discovery of scalp metastasis is 9 months (**Table 1**). Unfortunately, this patient noticed the subcutaneous mass only 2 months after the initial surgery, and the mass had rapidly grown over 5 cm within 1 month (**Figures 1D, E**). One possible reason is that the patient declined further treatments after the first surgery. On the other hand, the extracranial tumor was tightly adhered to the temporalis, which may allow it to get dual blood supply from extracranial temporalis and the intracranial tumor. This was also confirmed in imaging and immunohistochemical analysis of the patient. CT perfusion indicated that the blood supply was lower in the core region of the intracranial tumor compared to the core region of the extracranial tumor (**Figure 1B**). The FA and ADC are the main parameters of diffusion tensor imaging, in which FA is the proportion of FA of free water molecules in the total diffusion tensor and can reflect the diffusion limitation, and ADC can reflect the diffusion activity of water molecules (26, 27). Overall, higher values of ADC in tumor patients represent an impairment because of uncontrolled/less restricted diffusion, similar to low FA values (28). In this study, we also found extracranial tumor areas that have low ADC showing larger FA values than intracranial tumor areas (**Figure 1C**). Although ADC/FA values could be affected by various factors, we showed the ADC/FA maps of this patient to better reflect the radiographic features of intracranial and extracranial tumors.

However, similar results were not obtained from animal experiments. This might be explained by the following reasons. First, there are no muscle or other blood-rich tissues under the parietal scalp in mice. We observed no obvious adhesions between the extracranial tumor and surrounding tissues. The blood supply of the extracranial tumor originated from the intracranial tissue through the drilled channel. The necrotic areas were identified in both intracranial and extracranial tumors. However, the signs of necrosis could be seen in the intracranial tumor core but not in the extracranial tumor based on the patient HE staining. Second, the extracranial tumor of mice was developed from the intracranial tumor within a few days. A possible reason for this is that there were no indistinguishable histological characteristics between intracranial and extracranial tumors. However, in this patient, there is enough time to change for the TME of the extracranial tumor. In short, mice were not able to model the TME heterogeneity of patients; nonetheless, they suggest that glioma cells could also rapidly spread to subcutaneous space *via* the channel of craniotomy defect in the mouse model. Actually, it is one of the main limitations of this work.

The IHC results of this patient are consistent with CT perfusion findings, which reflect that the blood supply of the extracranial tumor is significantly more enriched than the intracranial tumor. Previous research fully affirmed that MMP2 and MMP9 play important roles in the infiltrative growth of gliomas (29). In this study, we found that both MMP2 and MMP9 expressions are significantly higher (*p* < 0.001) in the intracranial tumor. This might be due to the fact that the hypoxic microenvironment of the intracranial tumor causes glioma cells to secrete more MMP2/9 and thus acquire stronger invasion ability.

GBMs with extra-neural metastases have a poor prognosis; the median overall survival from diagnosis of metastasis was about 6 months (30). The female patient whom we reported further declined any subsequent treatments after the second surgery and died of disease progression 5 months later. We analyzed the survival time of GBM patients with scalp involvement, and the median survival time from scalp metastasis to death was only 4 months (**Table 1**). This may be due to the fact that some patients did not receive standard treatments or had other multiple-site metastases in addition to scalp metastasis.

Out of 24 patients reviewed, there are 8 patients present with other multiple-site metastases, such as lungs, liver, and lymph nodes (**Table 1**). The number may actually be higher, since imaging examinations in areas of the body other than the head were not performed in some patients. More distant metastases may result from the invasion of lymphatics and blood vessels by dura or scalp extension through the surgical defect (12). Some scholars think that positron emission tomography (PET)-CT is a systemic examination and could be used to detect the presence of systemic metastases (14). Some surgical strategies are believed to prevent extracranial metastases resulting from intraoperative seeding, including immediate postoperative prophylactic craniospinal irradiation, cranial defect restoration, and changing instruments between intradural and extradural segments of the operation (8).

## Conclusion

This is the first study to contrast the TME of an intracranial tumor and a scalp metastatic tumor. Craniectomy is a direct cause of subcutaneous metastasis in patients with GBM. Appropriate protective strategies are recommended during surgery to reduce the risk of extracranial metastasis. Moreover, imaging examinations of other sites for systemic screening are also recommended to look for metastases outside the brain when GBM invades the scalp or metastasizes to it.

## Data availability statement

The original contributions presented in the study are included in the article/**Supplementary Material**. Further inquiries can be directed to the corresponding author.

## Ethics statement

The animal study was reviewed and approved by The Animal Ethical and Welfare Committee of Zhejiang Provincial People’s Hospital. The studies involving human participants were reviewed and approved by The Medical Ethics Committee of Zhejiang Provincial People's Hospital. The patients/participants provided their written informed consent to participate in this study. Written informed consent was obtained from the individual(s) for the publication of any potentially identifiable images or data included in this article.

## Author contributions

Conception and design: SH and HL. Case collection: FW, JZ and HZ. Writing, revision of the manuscript: FW, JD, and HL. Clinical follow-up: JJ, XY and XG. Animal experiment: FW, HL, and NW. Funding: SH. All authors contributed to the article and approved the submitted version.

## Supplementary material

Supplementary Figure 1 The serial perioperative imaging and histopathological characteristics at first surgery. **(A)** Pre-operative axial computer tomography scan images showing the temporal tumor. **(B)** The postoperative magnetic resonance imaging (3 days after surgery). **(C)** Hematoxylin and eosin stains of the original tumor show tumor with necrosis and glomerular vascular proliferation. Scale bar = 100 μm.

Supplementary Figure 2 The timeline of therapy and disease status of the patient.

## References

[1] Stupp R Mason WP van den Bent MJ Weller M Fisher B Taphoorn MJ . Radiotherapy plus concomitant and adjuvant temozolomide for glioblastoma. N Engl J Med (2005) 352(10):987–96. doi: 10.1056/NEJMoa043330 15758009

[2] Lun M Lok E Gautam S Wu E Wong ET . The natural history of extracranial metastasis from glioblastoma multiforme. J Neurooncol (2011) 105(2):261–73. doi: 10.1007/s11060-011-0575-8 21512826

[3] Nakib CE Hajjar R Zerdan MB Darwish H Zeidan Y Alame S . Glioblastoma multiforme metastasizing to the skin, a case report and literature review. Radiol Case Rep (2022) 17(1):171–5. doi: 10.1016/j.radcr.2021.10.029 PMC8593264 34815821

[4] Varghese F Bukhari AB Malhotra R De A . IHC profiler: an open source plugin for the quantitative evaluation and automated scoring of immunohistochemistry images of human tissue samples. PloS One (2014) 9(5):e96801. doi: 10.1371/journal.pone.0096801 24802416 PMC4011881

[5] Seyed Jafari SM Hunger RE . IHC optical density score: A new practical method for quantitative immunohistochemistry image analysis. Appl Immunohistochem Mol Morphol (2017) 25(1):e12–e3. doi: 10.1097/PAI.0000000000000370 27093452

[6] Capion T Hauerberg J Broholm H Muhic A . Multiple extracranial metastases from primary gliosarcoma in a patient with two previous different primary cancers. Case Rep Oncol Med (2019) 2019:7849616. doi: 10.1155/2019/7849616 31565453 PMC6745105

[7] Perez-Bovet J Rimbau-Munoz J . Glioblastoma multiforme metastases to the masticator muscles and the scalp. J Clin Neurosci (2018) 53:237–9. doi: 10.1016/j.jocn.2018.04.021 29685418

[8] Forsyth TM Bi WL Abedalthagafi M Dunn IF Chiocca EA . Extracranial growth of glioblastoma multiforme. J Clin Neurosci (2015) 22(9):1521–3. doi: 10.1016/j.jocn.2015.03.018 25956620

[9] Anghileri E Castiglione M Nunziata R Boffano C Nazzi V Acerbi F . Extraneural metastases in glioblastoma patients: Two cases with YKL-40-positive glioblastomas and a meta-analysis of the literature. Neurosurg Rev (2016) 39(1):37–45; discussion -6. doi: 10.1007/s10143-015-0656-9 26212701

[10] Jusue Torres I Jerez Fernandez P Ortega Zufiria J Rodriguez Barbero JM . Skin spread from an intracranial glioblastoma: Case report and review of the literature. BMJ Case Rep (2011) 2011. doi: 10.1136/bcr.09.2011.4858 PMC3207803 22675113

[11] Saad AG Sachs J Turner CD Proctor M Marcus KJ Wang L . Extracranial metastases of glioblastoma in a child: Case report and review of the literature. J Pediatr Hematol Oncol (2007) 29(3):190–4. doi: 10.1097/MPH.0b013e31803350a7 17356401

[12] Ginat DT Kelly HR Schaefer PW Davidson CJ Curry W . Recurrent scalp metastasis from glioblastoma following resection. Clin Neurol Neurosurg (2013) 115(4):461–3. doi: 10.1016/j.clineuro.2012.05.038 22727368

[13] Luan XZ Wang HR Xiang W Li SJ He H Chen LG . Extracranial multiorgan metastasis from primary glioblastoma: A case report. World J Clin Cases (2021) 9(33):10300–7. doi: 10.12998/wjcc.v9.i33.10300 PMC8638031 34904103

[14] Liu J Shen L Tang G Tang S Kuang W Li H . Multiple extracranial metastases from glioblastoma multiforme: a case report and literature review. J Int Med Res (2020) 48(6):300060520930459. doi: 10.1177/0300060520930459 32552287 PMC7303784

[15] Schou Noroxe D Regner Michaelsen S Broholm H Moller S Skovgaard Poulsen H Lassen U . Extracranial metastases in glioblastoma-two case stories. Clin Case Rep (2019) 7(2):289–94. doi: 10.1002/ccr3.1980 PMC6389476 30847191

[16] Frade Porto N Delgado Fernandez J Garcia Pallero MLA Penanes Cuesta JR Pulido Rivas P Gil Simoes R . Subcutaneous tissue metastasis from glioblastoma multiforme: A case report and review of the literature. Neurocirugia (Astur Engl Ed) (2019) 30(3):149–54. doi: 10.1016/j.neucir.2018.03.005 29778285

[17] Nguyen B Samara J Lee A Fadia M Ngu C Pranavan G . A case of subgaleal metastasis from glioblastoma multiforme. Intern Med J (2018) 48(6):741–2. doi: 10.1111/imj.13818 29898273

[18] Senetta R Trevisan E Ruda R Benech F Soffietti R Cassoni P . Skin metastases of glioblastoma in the absence of intracranial progression are associated with a shift towards a mesenchymal immunophenotype: Report of two cases. Acta Neuropathol (2009) 118(2):313–6. doi: 10.1007/s00401-009-0543-y 19418061

[19] Mentrikoski M Johnson MD Korones DN Scott GA . Glioblastoma multiforme in skin: A report of 2 cases and review of the literature. Am J Dermatopathol (2008) 30(4):381–4. doi: 10.1097/DAD.0b013e31817532c4 18645311

[20] Schultz S Pinsky GS Wu NC Chamberlain MC Rodrigo AS Martin SE . Fine needle aspiration diagnosis of extracranial glioblastoma multiforme: Case report and review of the literature. Cytojournal (2005) 2:19. doi: 10.1186/1742-6413-2-19 16287502 PMC1325054

[21] Allan RS . Scalp metastasis from glioblastoma. J Neurol Neurosurg Psychiatry (2004) 75(4):559. 15026496 PMC1739022

[22] Jain N Mirakhur M Flynn P Choudhari KA . Cutaneous metastasis from glioblastoma. Br J Neurosurg (2005) 19(1):65–8. doi: 10.1080/02688690500081423 16147588

[23] Figueroa P Lupton JR Remington T Olding M Jones RV Sekhar LN . Cutaneous metastasis from an intracranial glioblastoma multiforme. J Am Acad Dermatol (2002) 46(2):297–300. doi: 10.1067/mjd.2002.104966 11807444

[24] Houston SC Crocker IR Brat DJ Olson JJ . Extraneural metastatic glioblastoma after interstitial brachytherapy. Int J Radiat Oncol Biol Phys (2000) 48(3):831–6. doi: 10.1016/s0360-3016(00)00662-3 11020581

[25] Carvalho PA Schwartz RB Alexander E 3rd, Loeffler JS Zimmerman RE Nagel JS . Extracranial metastatic glioblastoma: Appearance on thallium-201-chloride/technetium-99m-HMPAO SPECT images. J Nucl Med (1991) 32(2):322–4. 1846913

[26] Meoded A Faria AV Hartman AL Jallo GI Mori S Johnston MV . Cerebral reorganization after hemispherectomy: A DTI study. AJNR Am J Neuroradiol (2016) 37(5):924–31. doi: 10.3174/ajnr.A4647 PMC7218754 26767710

[27] Caravan I Ciortea CA Contis A Lebovici A . Diagnostic value of apparent diffusion coefficient in differentiating between high-grade gliomas and brain metastases. Acta Radiol (2018) 59(5):599–605. doi: 10.1177/0284185117727787 28835111

[28] Rosenstock T Hani L Grittner U Schlinkmann N Ivren M Schneider H . Bicentric validation of the navigated transcranial magnetic stimulation motor risk stratification model. J Neurosurg (2022) 136(4):1194–206. doi: 10.3171/2021.3.JNS2138 34534966

[29] Tabouret E Boudouresque F Farina P Barrie M Bequet C Sanson M . MMP2 and MMP9 as candidate biomarkers to monitor bevacizumab therapy in high-grade glioma. Neuro Oncol (2015) 17(8):1174–6. doi: 10.1093/neuonc/nov094 PMC4490881 26142442

[30] Pietschmann S von Bueren AO Kerber MJ Baumert BG Kortmann RD Muller K . An individual patient data meta-analysis on characteristics, treatments and outcomes of glioblastoma/ gliosarcoma patients with metastases outside of the central nervous system. PloS One (2015) 10(4):e0121592. doi: 10.1371/journal.pone.0121592 25860797 PMC4393116

