# Supplementary figures and images for: Rapid progression of subcutaneous glioblastoma: A case report and literature review
Source: Front Oncol. 2023 Jan 25;13:935944. doi: 10.3389/fonc.2023.935944 (PMC9905810; doi:10.3389/fonc.2023.935944)

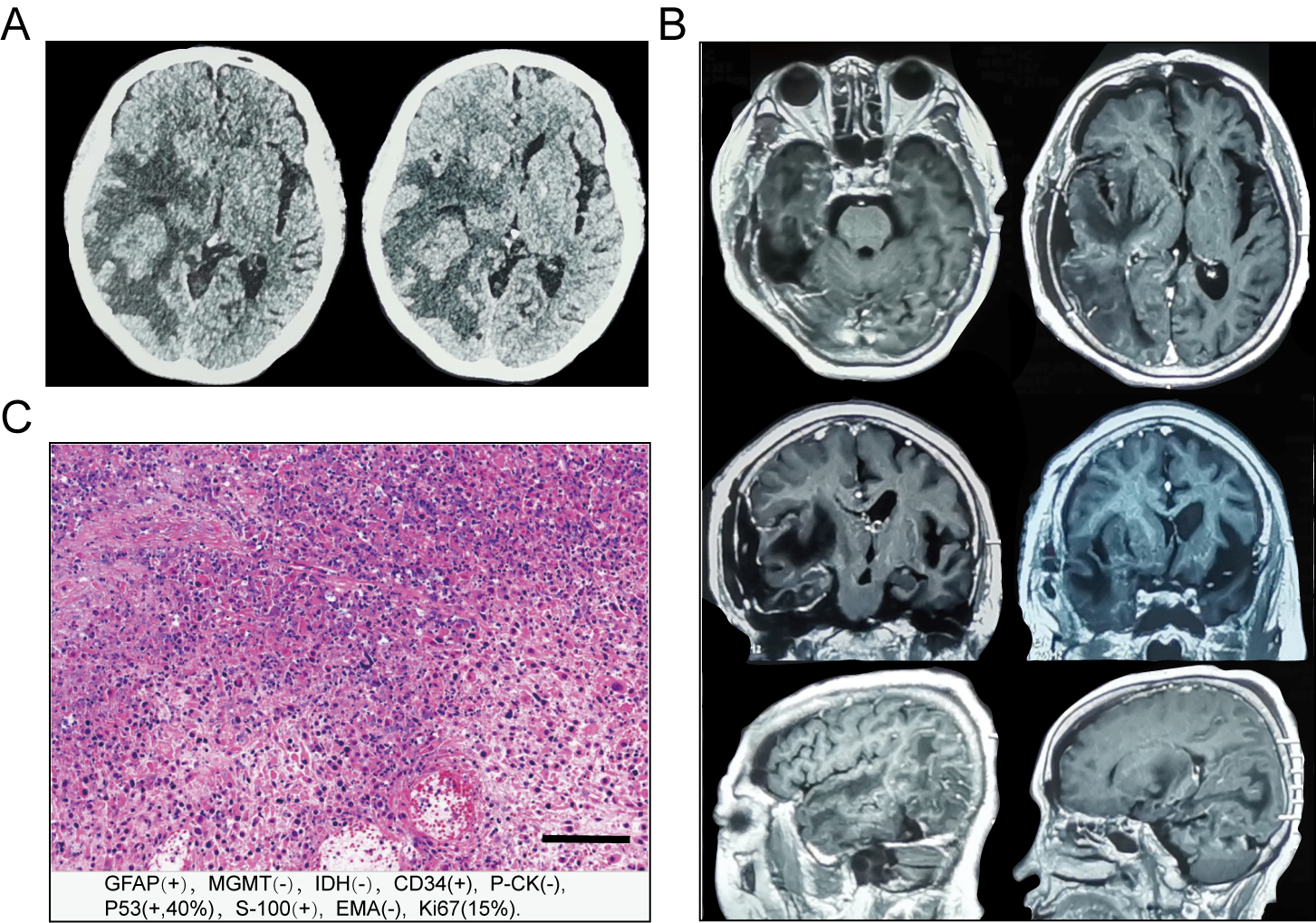

Supplement: Supplementary Figure 1 — The serial perioperative imaging and histopathological characteristics at first surgery. (A) Pre-operative axial computer tomography scan images showing the temporal tumor. (B) The postoperative magnetic resonance imaging (3 days after surgery). (C) Hematoxylin and eosin stains of the original tumor show tumor with necrosis and glomerular vascular proliferation. Scale bar = 100 μm. [file Image_1.tif]

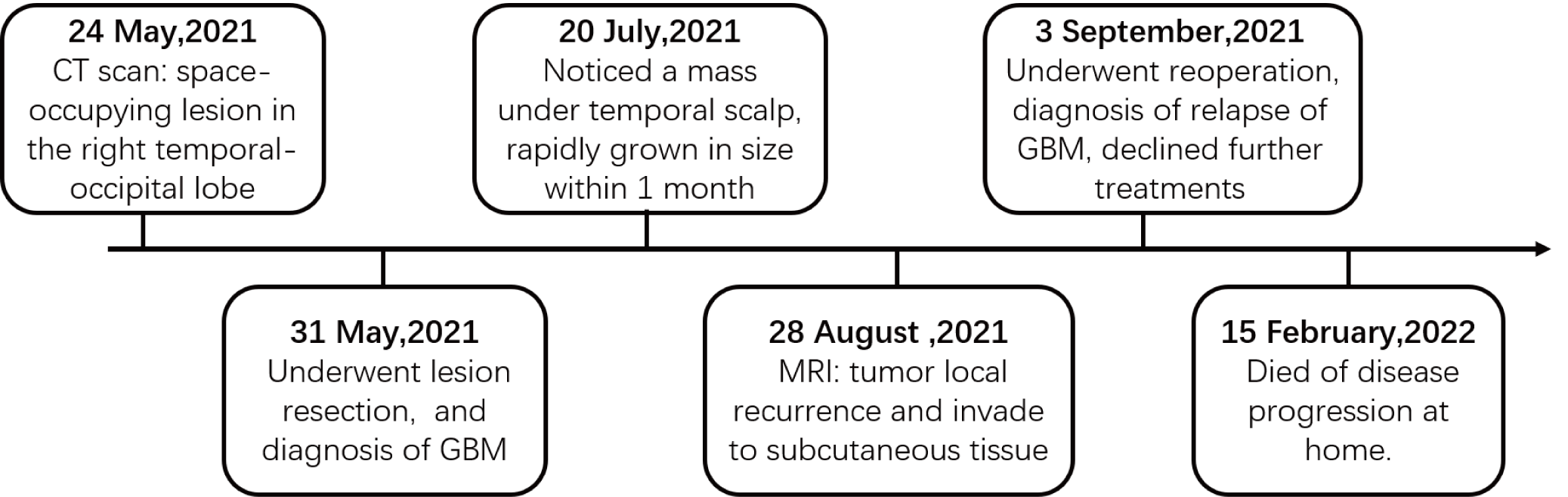

Supplement: Supplementary Figure 2 — The timeline of therapy and disease status of the patient. [file Image_2.tif]
